# Novel Clinical Evidence of an Association between Homocysteine and Insulin Resistance in Patients with Hypothyroidism or Subclinical Hypothyroidism

**DOI:** 10.1371/journal.pone.0125922

**Published:** 2015-05-04

**Authors:** Ning Yang, Zhi Yao, Li Miao, Jia Liu, Xia Gao, Hui Fan, Yanjin Hu, Heng Zhang, Yuan Xu, Aijuan Qu, Guang Wang

**Affiliations:** 1 Department of Endocrinology, Beijing Chaoyang Hospital, Capital Medical University, Beijing, People’s Republic of China; 2 Department of Physiology and Pathophysiology, School of Basic Medical Science, Capital Medical University, Beijing, People’s Republic of China; Medical University Innsbruck, AUSTRIA

## Abstract

**Objective:**

Hypothyroidism (HO) can induce significant metabolic dysfunction and increase cardiovascular disease risk. In the present study, we investigated the relationship between homocysteine (Hcy) and insulin resistance (IR) in patients with HO or subclinical hypothyroidism (SHO).

**Methods:**

A total of 270 subjects were enrolled. All subjects were divided into the following three groups: HO, SHO and control. Plasma levels of Hcy were measured, and each patient’s homeostatic index of insulin resistance (HOMA-IR) was calculated. Statistical analyses were carried out to evaluate the correlations among groups and to determine the predictors of IR in patients with HO or SHO.

**Results:**

The HOMA-IR value was significantly higher in the HO group than in the SHO and control groups. Plasma levels of Hcy were markedly increased in the HO group compared with those of the SHO group and controls. In addition, plasma levels of Hcy were positively correlated with the HOMA-IR values in both the HO and SHO groups. Multiple linear regression models showed that plasma levels of Hcy and free thyroxine (FT4) were the only predictors of HOMA-IR in patients with HO or SHO.

**Conclusions:**

Plasma levels of Hcy and HOMA-IR were increased in patients with HO or SHO. Our results suggest that HO and SHO may increase the risk for atherogenesis and cardiovascular disease by increased IR. The increased IR induced by hyperhomocysteinemia in patients with HO or SHO may partially explain this adverse effect.

## Introduction

Hypothyroidism (HO) and subclinical hypothyroidism (SHO), the two most common endocrine disorders, can induce metabolic dysfunction and increase cardiovascular disease risk [1,2]. HO is a thyroid hormone deficiency and can be due to primary disease of the thyroid gland itself. SHO is defined as a serum thyrotrophin (TSH) concentration above the statistically defined upper limit of the reference range when serum free thyroxine (FT4) and free tri-iodothyronine (FT3) concentrations are within their reference ranges [3,4]. In patients with HO or SHO, dyslipidemia may be partially responsible for the high risk of vascular disease [5,6]. Recently, several studies have demonstrated the presence of insulin resistance (IR) not only in HO but also in SHO patients [7,8]. IR disturbs the insulin pathway in target organs such as the liver, muscles, and adipose tissue. This disturbance affects glucose metabolism, lipogenesis and adipokine production. IR induced vascular endothelium dysfunction plays an important role in the initiation and progression of atherosclerosis and significantly increases the risk for coronary heart disease [9,10].

Previous studies have demonstrated that hyperhomocysteinemia (HHcy) is an independent risk factor for atherosclerosis [11,12]. HHcy increases cardiovascular disease by various mechanisms including endothelial dysfunction, oxidative stress, endoplasmic reticulum stress, smooth muscle cell proliferation and platelet aggregation [13–16]. Recent investigations found that Hcy levels were increased in patients with HO [17,18]. The increased Hcy levels may be associated with IR through the induction of resistin expression and secretion by adipocytes [19–21]. Our previous study demonstrated that HHcy may contribute to atherogenesis by enhancing the responsiveness of monocytes to inflammatory stimuli and promote IR by inducing endoplasmic reticulum stress in adipose tissue [22]. Our studies also showed that HHcy can damage coronary artery endothelial function in hyperhomocysteinemic patients [23,24]. However, the underlying effects of Hcy on IR during HO remain unclear. In the present study, we investigate whether Hcy can aggravate IR in patients with HO or SHO.

## Materials and Methods

### Subjects

This study initially enrolled 253 outpatients who were treated in our clinic from Jan. 2013 to Dec. 2013. SHO is characterized by a serum TSH above the upper reference limit in combination with a normal FT4. This designation is only applicable when thyroid function has been stable for weeks or more, the hypothalamic-pituitary-thyroid axis is normal, and there is no recent or ongoing severe illness. An elevated TSH, usually above 10 mIU/L, in combination with a subnormal FT4 characterizes overt HO [25]. Exclusion criteria were patients with cardiovascular disease, hypertension, diabetes mellitus or impaired glucose tolerance, renal diseases or other endocrine diseases. Therefore, 73 patients were excluded. The final study cohort included 180 patients of those initially enrolled, which included 78 patients with HO and 102 patients with SHO. All patients did not received any treatment. The control group included 90 normal, non-hypothyroid volunteers who were seeking routine medical care at the physical examination center of Beijing Chaoyang Hospital. The study protocol was designed according to the Declaration of Helsinki guidelines and approved by the Medical Ethics Committee of Beijing Chaoyang Hospital. Written informed consent was obtained from all patients.

### Sample collection

Basic demographic information (i.e., age, sex, body height and weight) was collected from each patient. Subjects wore only underwear for height and weight measurements, which were assessed to the nearest 0.5 cm and 0.1 kg, respectively, by a well-trained examiner. Body mass index (BMI) was calculated as the weight in kilograms divided by the height in meters squared. After an overnight fast, a blood sample was collected from the peripheral vein of each patient and subject to a routine analysis, consisting of Hcy, fasting blood-glucose (FPG), total cholesterol (CHOL), high-density lipoprotein cholesterol (HDL-C), low-density lipoprotein cholesterol (LDL-C), triglyceride (TG), hemoglobin A1c (HbA1c), fasting insulin (FINS), FT3, FT4, and TSH measurements.

### Measurement of plasma Hcy levels

Plasma Hcy concentrations were determined by enzymatic cycling assay-based quantification using the corresponding kits from Baiding Biotech (Beijing, China) according to the manufacturer’s instructions. The normal reference value is less than 15 μmol/l [26,27].

### Measurement of FPG, blood lipid and thyroid function indexes, FINS, and HbA1c

FPG, CHOL, HDL-C, LDL-C, and TG were determined using a Dade-Behring Dimension RXL Autoanalyzer (Dade Behring Diagnostics, Marburg, Germany). The reference intervals for CHOL, HDL-C, LDL-C and TG were 3.62–5.7 mmol/l, 1.03–1.55 mmol/l, 1.81–3.36 mmol/l and 0.56–2.26 mmol/l, respectively.

FT3, FT4 and TSH were determined by electrochemiluminescence immunoassay (ECLIA) using an Abbott Architect i2000 (Abbott Diagnostics, Abbott Park, IL, USA). The reference intervals for FT3, FT4 and TSH were 1.71–3.71 pg/ml, 0.7–1.48 ng/dl and 0.35–4.94 μIU/ml, respectively.

FINS was measured on a Beckman Access 2 (Fullerton, CA, USA), and the reference interval was 1.9–23 mIU/ml. HbA1c was estimated by high-performance liquid chromatography using the HLC-723G7 analyzer (Tosoh Corporation, Japan) with a reference interval of 4–6%.

### Evaluation of insulin resistance

The homeostatic index of insulin resistance (HOMA-IR) was used to evaluate IR because it is well-known for assessing IR across a wide range of values and is well correlated with insulin-mediated glucoseuptake as calculated by euglycemic glucose clamp. The following formula was used to calculate HOMA-IR: fasting glucose (mmol/l) x fasting insulin (mU/ml)/22.5 [28].

### Statistical analyses

All statistical analyses were performed using the Statistical Package for the Social Sciences software package (Version 17.0, SPSS Inc, Chicago, IL) to identify significant effects between the patient groups and corresponding controls. Because HOMA-IR did not follow a normal distribution, comparisons between groups were carried out with Mann-Whitney U or Kruskal-Wallis H tests. Values were expressed as medians (25th and 75th percentiles), and quantitative data were presented as the mean ± standard deviation (SD). Comparisons between groups were performed using independent-samples or one-way ANOVA. Spearman’s rank correlation was used to assess the relationship between HOMA-IR and other variables. All tests were two-tailed and *p* values less than 0.05 were considered statistically significant.

## Results

### Clinical characteristics of subjects

**Fig 1:**
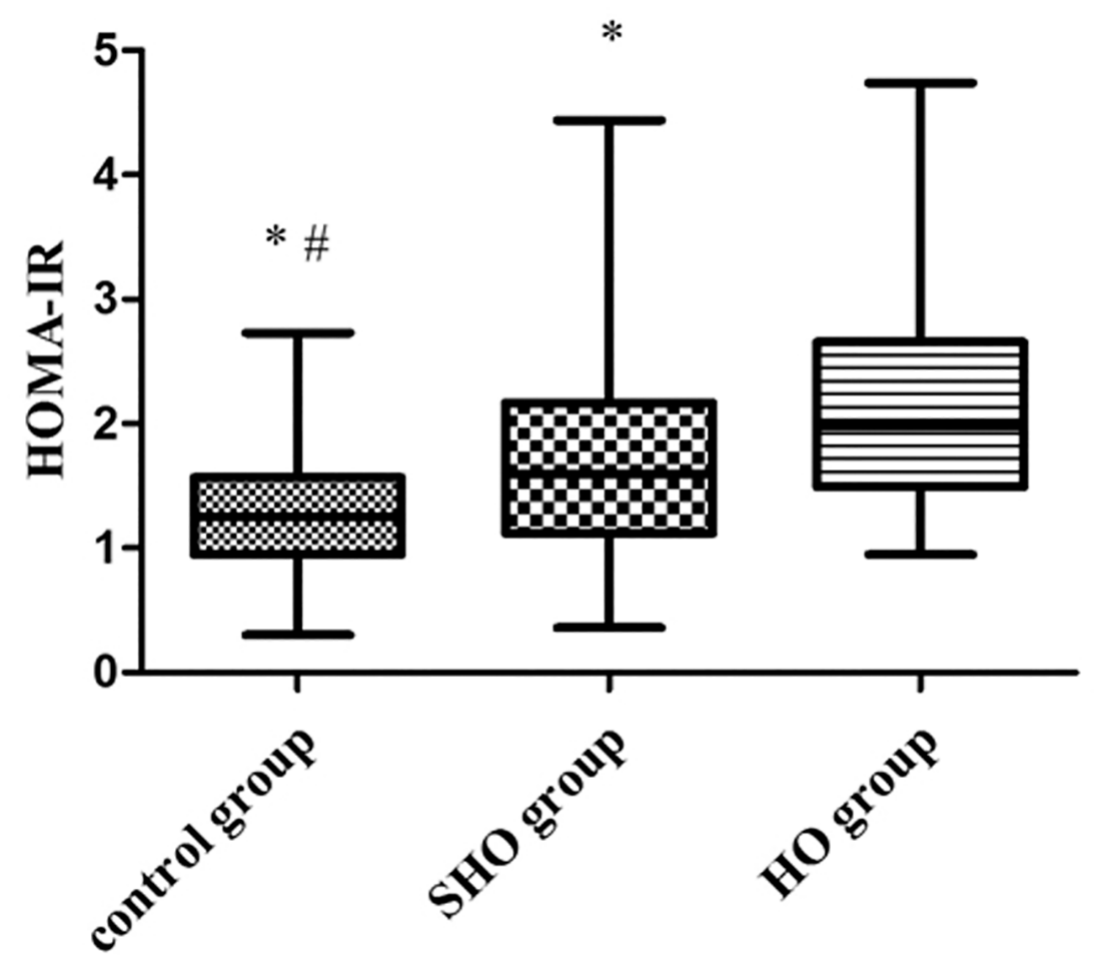
HOMA-IR values in study subjects, n = 90 in control group, n = 102 in SHO group and n = 78 in HO group. Values were expressed as medians and ranges.*p<0.01 vs. HO group, #*p*<0.01 vs. SHO group.

**Fig 2:**
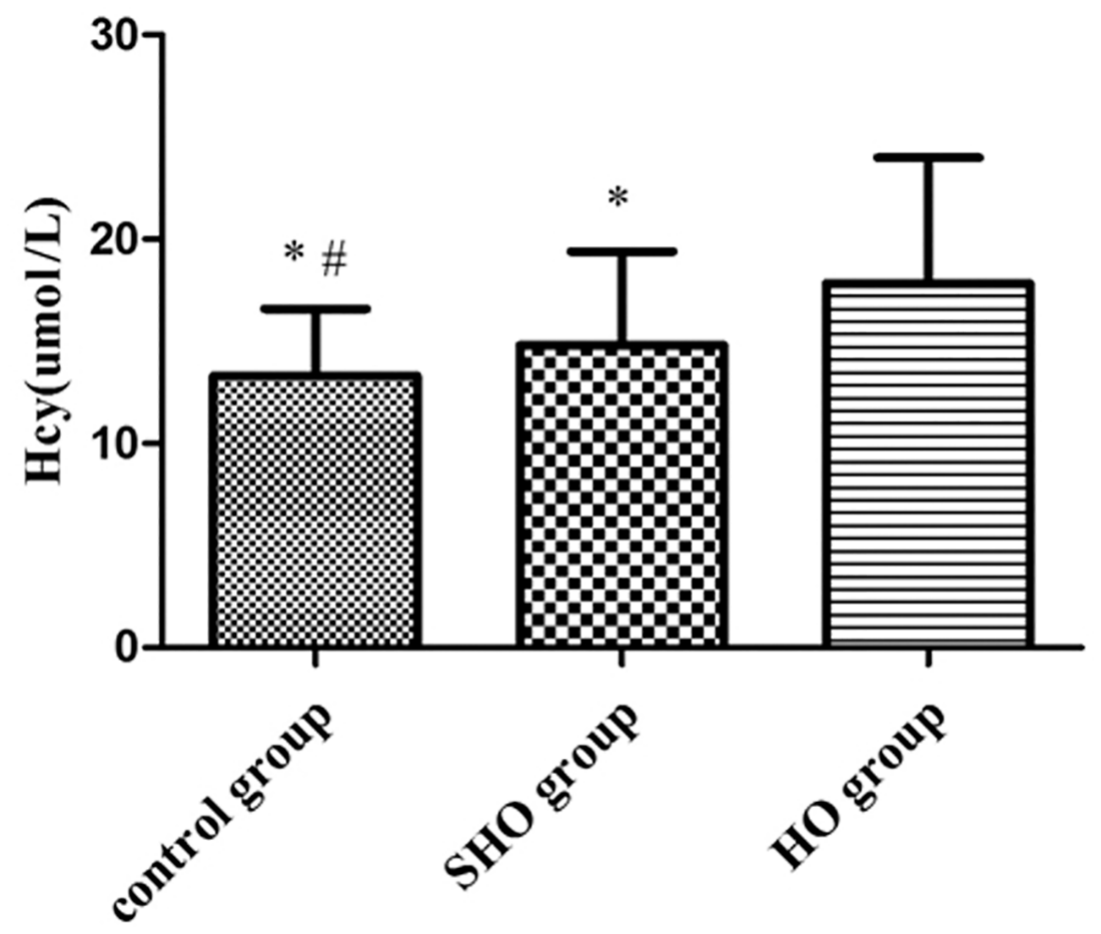
Plasma levels of Hcy in study subjects, n = 90 in control group, n = 102 in SHO group and n = 78 in HO group. Values were expressed as the means ± SD. *p<0.01 vs. HO group, #*p*<0.05 vs. SHO group.

The characteristics and laboratory examination values of the subjects are summarized in Table 1. The incidence of HO or SHO in females is significantly higher than in males. CHOL, HDL-C, LDL-C and TG values in the HO group were significantly higher than those in the SHO and control groups, although there was no difference between the SHO and control groups (CHOL: 6.25 ± 1.83 vs. 5.08 ± 1.23 and 4.88 ± 0.93 mmol/l in HO, SHO and control groups, respectively; HDL-C: 1.72 ± 0.48 vs. 1.52 ± 0.33 and 1.57 ± 0.34 mmol/l, LDL-C: 3.61 ± 1.17 vs. 2.99 ± 0.92 and 2.84 ± 0.81 mmol/l; TG: 1.69 ± 1.30 vs. 1.34 ± 0.73 and 1.24 ± 0.72 mmol/l; all *p*<0.05). A significant increase in FINS values was observed in patients with HO compared to those in the SHO and control groups (9.56 ± 3.62 vs. 7.55 ± 2.99 vs. 5.89 ± 2.11 mU/ml, all *p*<0.05). HOMA-IR values were significantly higher in the HO group than in the SHO group and controls [1.99(1.50 to 2.65) vs. 1.60(1.12 to 2.17) vs. 1.25(0.95 to 1.57), all *p*<0.01] (Fig 1). Furthermore, plasma Hcy levels were significantly higher in the HO group than in the SHO group and controls (17.85 ± 6.14 vs. 14.81 ± 4.57 vs. 13.31 ± 3.28 μmol/l, all *p*<0.05) (Fig 2). There was no significant difference among all three groups with regards to age, BMI, FPG, or HbA1c levels.

**Table 1:** General information and clinical characteristics of subjects.

	Control group (n = 90)	SHO (n = 102)	HO (n = 78)	*p-value*
Sex (M/F)	23/67	11/91†	7/71†	0.003**
Age,years	46.76 ± 11.11	45.60 ± 13.91	43.82 ± 14.40	0.354
BMI, km/m^2^	24.16 ± 3.34	24.41 ± 3.43	24.70 ± 3.38	0.593
FPG, mmol/l	5.07 ± 0.43	5.16 ± 0.66	5.09 ± 0.67	0.541
CHOL, mmol/l	4.88 ± 0.93	5.08 ± 1.23	6.25 ± 1.83† ‡	<0.001**
HDL-C, mmol/l	1.57 ± 0.34	1.52 ± 0.33	1.72 ± 0.48† ‡	0.002**
LDL-C, mmol/l	2.84 ± 0.81	2.99 ± 0.92	3.61 ± 1.17† ‡	<0.001**
TG, mmol/l	1.24 ± 0.72	1.34 ± 0.73	1.69 ± 1.30† ‡	0.007**
HbA1c, %	5.56± 0.40	5.62 ± 0.51	5.72 ± 0.42	0.081
FINS,mU/ml	5.89 ± 2.11	7.55 ± 2.99†	9.56 ± 3.62† ‡	<0.001**
HOMA-IR	1.25 (0.95–1.57)	1.60 (1.12–2.17) †	1.99 (1.50–2.65) † ‡	<0.001**
Hcy, μmol/l	13.31 ± 3.28	14.81 ± 4.57†	17.85 ± 6.14† ‡	<0.001**

Summary of the clinical characteristics and laboratory results of the study participants in 90 control subjects, 102 patients with SHO and 78 patients with HO. Data were expressed as the means ± SD. HOMA-IR values were given as medians and ranges. BMI: body mass index; FPG: fasting plasma glucose; CHOL: total cholesterol; HDL-C: high-density lipoprotein cholesterol; LDL-C: low-density lipoprotein cholesterol; TG: triglyceride; HbA1c: glycosylated hemoglobin; HOMA-IR: homeostatic index of insulin resistance; Hcy:homocysteine.

*p<0.05,

**p<0.01, significantly different among three groups;

^†^ *p*<0.05, significantly different compared with control group;

^‡^ *p*<0.05, significantly different compared with SHO group.

### Correlation between HOMA-IR and plasma levels of Hcy, blood lipid, and thyroid function indexes

**Fig 3:**
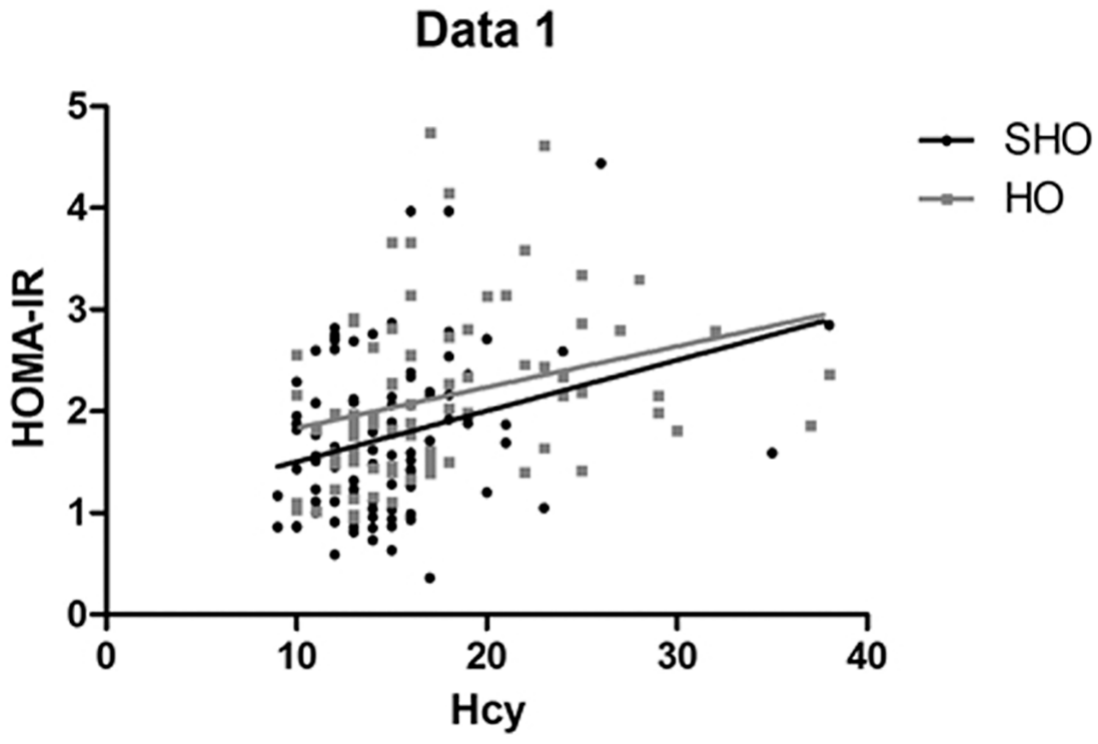
Correlation between HOMA-IR and plasma levels of Hcy in the SHO (n = 102) or HO (n = 78) groups. HOMA-IR was positively correlated with Hcy levels in the SHO (*r* = 0.23, *p*<0.05) and HO groups (*r* = 0.42, *p*<0.01).

Table 2 shows that HOMA-IR was positively correlated with LDL-C (*r* = 0.218, *p*<0.05) and negatively correlated with FT4 (*r* = -0.232, *p*<0.05) in patients with SHO. In the HO group, HOMA-IR was negatively correlated with FT3 (*r* = -0.338, *p*<0.01) and FT4 (*r* = -0.384, *p*<0.01) and positively correlated with TSH (*r* = 0.267, *p*<0.05). A significant positive correlation was observed between HOMA-IR and Hcy in the SHO (*r* = 0.225, *p*<0.05) and HO groups (*r* = 0.419, *p*<0.01) (Fig 3).

**Table 2:** Correlation between HOMA-IR and plasma levels of Hcy, blood lipid, and thyroid function indexes in controls and patients with HO or SHO.

	Control group (n = 90)		SHO (n = 102)		HO (n = 78)	
	*r*	*p-value*	*r*	*p-value*	*r*	*p-value*
Age, year	-0.014	0.894	-0.042	0.674	-0.007	0.955
BMI, km/m^2^	0.171	0.128	0.135	0.175	0.008	0.947
CHOL, mmol/l	0.041	0.700	0.158	0.112	0.120	0.302
HDL-C, mmol/l	-0.110	0.303	-0.107	0.283	-0.002	0.983
LDL-C, mmol/l	0.083	0.439	0.218	0.028*	0.127	0.276
TG, mmol/l	0.122	0.252	0.111	0.267	0.129	0.266
FT3, pg/ml	-0.129	0.556	-0.142	0.154	-0.338	0.002**
FT4, ng/dl	-0.112	0.610	-0.232	0.019*	-0.384	0.001**
TSH, μIU/ml	0.307	0.154	0.149	0.135	0.267	0.018*
Hcy, μmol/l	-0.028	0.794	0.225	0.010*	0.419	<0.001**

Spearman's rank correlation was used to assess the correlation between HOMA-IR and plasma levels of Hcy, blood lipid, and thyroid function indexes in the three groups. FT3: free tri-iodothyronine; FT4: free thyroxine, TSH: thyroid-stimulating hormone.

*p<0.05,

**p<0.01.

### Linear regression analysis showing factors associated with HOMA-IR

Observations from the multiple linear regression analysis in Table 3, after adjustment for age, BMI, blood lipid, thyroid function indexes and Hcy, showed that plasma levels of Hcy and FT4 were the only significant predictors of HOMA-IR in patients with HO or SHO (OR = 0.27 and -0.23, respectively, all *p*<0.01).

**Table 3:** Linear regression analysis showing factors associated with HOMA-IR in patients with HO and SHO.

	Beta	Std. error	Beta	t	*p-value*
Hcy	0.04	0.01	0.27	3.76	<0.001
FT4	-0.72	0.23	-0.23	-3.16	0.002

## Discussion

IR plays a key role in the pathogenesis of atherosclerosis, from the initiation and progression of disease to the formation of clinically significant plaques and coronary artery disease, even in patients without diabetes mellitus [9–11]. Several investigations demonstrated that HO and SHO patients exhibited IR that was associated with cardiovascular disease [1,2,7,8]. This suggests that previously unappreciated factors may be involved in the link between IR and atherosclerosis in patients with HO and SHO. The etiology of IR in HO is poorly understood and likely multifactorial. Some animal studies showed the excess secretion of growth hormone may contribute to IR in some hypothyroid dogs, but this has not yet been documented in clinical cases [29]. Our study points to plasma Hcy levels as one potential factor that may contribute to IR. Hcy was increased in hypothyroid patients [17,18], and elevated Hcy levels are toxic to vascular endothelium through the induction of endothelial dysfunction and the development of atherosclerosis independent of standard coronary artery disease risk factors [30–32]. The relationship between IR and Hcy was reported in patients with polycystic ovary syndrome and patients with hypertension [19,20,33]. Our previous study demonstrated that Hcy might act as an atherogenic factor by promoting the production of chemokines, reactive oxygen species and oxidized LDL-C, thus enhancing the progression of cardiovascular disease [34]. Our previous studies also showed that Hcy can induce coronary endothelial injury in hyperhomocysteinemic patients and can also promote chemokine expression and IR by inducing endoplasmic reticulum stress in human monocytes and adipose tissue [12]. In this study, we provide data regarding the positive correlation between IR and Hcy in patients with HO and SHO for the first time.

We observe that subjects in the HO group had higher CHOL, HDL-C, LDL-C, and TG than did subjects in the SHO and control groups. In our study, FINS, HOMA-IR and Hcy were significantly higher in the HO group than in the SHO and control groups. Increased HOMA-IR suggested that IR was present in patients with HO and SHO [7,8]. Some studies have shown that the presence of IR due to impaired glucose disposal in peripheral tissues rapidly increases glucose disposal rates in patients with HO and SHO [8,35]. These data are consistent with previous studies that have reported an increased cardiovascular risk in these conditions [36,37]. Hcy was significantly higher in the HO group than in the SHO and control groups. Our results were consistent with the findings of the previous study [17,18].

Our results showed a positive correlation between HOMA-IR and Hcy levels in the HO and SHO groups. Little is known about the relationship between Hcy and IR in patients with HO and SHO. A recent animal study suggests that insulin affects the activities of the key enzymes involved in Hcy metabolism [38]. Conversely, some studies showed that folate treatment improved IR along with decreasing Hcy and showed that levothyroxine replacement therapy in patients with HO reduced the plasma levels of Hcy and improved insulin resistance [39,40], adding new data on the potential relationship between IR and Hcy levels. HOMA-IR was negatively correlated with FT3 and FT4 in the HO group and with FT4 in the SHO group, suggesting that the lower plasma FT3 and FT4 levels promoted higher IR in tissues. T3 and insulin act synergistically during glucose homeostasis. Both hormones possess similar sites of action in the regulation of glucose metabolism at the cellular and molecular levels [41]. We hypothesize that reduced T3 could lead to impaired insulin-stimulated glucose disposal. Studies have shown that a low normal FT4 level was significantly positively correlated with increased IR [42]. Therefore, these data support the hypothesis that thyroid function is associated with IR [7,8].

From the regression analysis, it was clear that Hcy and FT4 were the significant predictors for HOMA-IR in patients with HO and SHO. Low FT4 levels were significantly associated with increased IR and consistent with increased cardiovascular risk [42]. HHcy and IR were both associated with endothelial dysfunction and cardiovascular disease. In our study, elevated plasma levels of Hcy promoted IR, which was consistent with our previous studies suggesting that HHcy was associated with IR [17,43]. We therefore postulate that HHcy induces IR in patients with HO and SHO.

Plasma levels of Hcy and HOMA-IR were increased in patients with HO or SHO. Our results suggest that HO and SHO may elevate the risk for atherogenesis and cardiovascular disease by promoting IR. This increased IR induced by HHcy in patients with HO or SHO may partially explain this adverse effect.
